# Validation of a Step Detection Algorithm during Straight Walking and Turning in Patients with Parkinson’s Disease and Older Adults Using an Inertial Measurement Unit at the Lower Back

**DOI:** 10.3389/fneur.2017.00457

**Published:** 2017-09-04

**Authors:** Minh H. Pham, Morad Elshehabi, Linda Haertner, Silvia Del Din, Karin Srulijes, Tanja Heger, Matthis Synofzik, Markus A. Hobert, Gert S. Faber, Clint Hansen, Dina Salkovic, Joaquim J. Ferreira, Daniela Berg, Álvaro Sanchez-Ferro, Jaap H. van Dieën, Clemens Becker, Lynn Rochester, Gerhard Schmidt, Walter Maetzler

**Affiliations:** ^1^Department of Neurology, University of Kiel, Kiel, Germany; ^2^Digital Signal Processing and System Theory, Faculty of Engineering, University of Kiel, Kiel, Germany; ^3^Center for Neurology, Department of Neurodegeneration, Hertie Institute for Clinical Brain Research (HIH), University of Tübingen, Tübingen, Germany; ^4^DZNE, German Center for Neurodegenerative Diseases, Tübingen, Germany; ^5^Institute of Neuroscience/Newcastle University Institute for Ageing, Clinical Ageing Research Unit, Campus for Ageing and Vitality, Newcastle University, Newcastle upon Tyne, United Kingdom; ^6^Department of Clinical Gerontology, Robert Bosch Hospital, Stuttgart, Germany; ^7^Department of Human Movement Sciences, MOVE Research Institute Amsterdam, VU University Amsterdam, Amsterdam, Netherlands; ^8^Clinical Pharmacology Unit, Instituto de Medicina Molecular, Lisbon, Portugal; ^9^Laboratory of Clinical Pharmacology and Therapeutics, Faculty of Medicine, University of Lisbon, Lisbon, Portugal; ^10^HM CINAC, Hospital Universitario HM Puerta del Sur, Móstoles, Madrid, Spain; ^11^Research Laboratory of Electronics, Massachusetts Institute of Technology, Cambridge, MA, United States; ^12^Newcastle upon Tyne Hospitals NHS Foundation Trust, Newcastle upon Tyne, United Kingdom

**Keywords:** accelerometer, gait analysis, home-like activities, older adults, Parkinson’s disease, turning

## Abstract

**Introduction:**

Inertial measurement units (IMUs) positioned on various body locations allow detailed gait analysis even under unconstrained conditions. From a medical perspective, the assessment of vulnerable populations is of particular relevance, especially in the daily-life environment. Gait analysis algorithms need thorough validation, as many chronic diseases show specific and even unique gait patterns. The aim of this study was therefore to validate an acceleration-based step detection algorithm for patients with Parkinson’s disease (PD) and older adults in both a lab-based and home-like environment.

**Methods:**

In this prospective observational study, data were captured from a single 6-degrees of freedom IMU (APDM) (3DOF accelerometer and 3DOF gyroscope) worn on the lower back. Detection of heel strike (HS) and toe off (TO) on a treadmill was validated against an optoelectronic system (Vicon) (11 PD patients and 12 older adults). A second independent validation study in the home-like environment was performed against video observation (20 PD patients and 12 older adults) and included step counting during turning and non-turning, defined with a previously published algorithm.

**Results:**

A continuous wavelet transform (cwt)-based algorithm was developed for step detection with very high agreement with the optoelectronic system. HS detection in PD patients/older adults, respectively, reached 99/99% accuracy. Similar results were obtained for TO (99/100%). In HS detection, Bland–Altman plots showed a mean difference of 0.002 s [95% confidence interval (CI) −0.09 to 0.10] between the algorithm and the optoelectronic system. The Bland–Altman plot for TO detection showed mean differences of 0.00 s (95% CI −0.12 to 0.12). In the home-like assessment, the algorithm for detection of occurrence of steps during turning reached 90% (PD patients)/90% (older adults) sensitivity, 83/88% specificity, and 88/89% accuracy. The detection of steps during non-turning phases reached 91/91% sensitivity, 90/90% specificity, and 91/91% accuracy.

**Conclusion:**

This cwt-based algorithm for step detection measured at the lower back is in high agreement with the optoelectronic system in both PD patients and older adults. This approach and algorithm thus could provide a valuable tool for future research on home-based gait analysis in these vulnerable cohorts.

## Introduction

Gait deficits are common in older adults (1) and Parkinson’s disease (PD) patients, even in early disease stages (2, 3). They are associated with an increased risk of falling and reduced quality of life (4, 5). Temporal step parameters are crucial in describing the quality of gait, e.g., to calculate the risk of future falls and response to treatment. Examples are variability of stride time and kinematics (6, 7), gait speed (8), and symmetry (9). Heel strike (HS) and toe off (TO) (10, 11) are critical events during a gait cycle because they define the beginning and the end of every stance and swing phase, respectively (12) and enable the calculation of all of the above parameters.

Several methods have been successfully utilized to extract time-related gait parameters, including optical systems (13, 14), instrumented walkways (12, 15), and treadmills (16). However, such equipment is expensive, restricted to specialized laboratories, and can thus not be applied in large populations and in home environments. This is a relevant drawback, as the home environment may be the most appropriate setting to capture and study gait related issues that are relevant to the patient’s daily functioning (17, 18), rather than constrained lab settings.

Wearable sensors, such as inertial measurement units (IMUs), are relatively cheap, light-weight, easy to use, and therefore a promising alternative approach for data collection in the home environment (19, 20). They are particularly useful for gait analysis, as shown in a relatively large number of studies, e.g., in healthy adults (21, 22) older adults (23), and patients with PD (24). However, for the use of such devices under medical conditions, a thorough validation of detection algorithms is necessary and must be performed in every single population presenting specific gait impairments (17). Furthermore, movement detection algorithms should be able to differentiate between gait episodes, such as straight walking and turning. Parameters like the number of steps during turning might be robust indicators of gait impairment in PD (25, 26). This differentiation could help detecting context-dependent gait deficits, as shown to occur in older adults with poor cognitive flexibility (27).

The algorithm development for IMUs is under constant improvement. Various step detection algorithms have been proposed, but their validation is limited to laboratory settings, and an extrapolation of the results to home-like environments is often lacking. Measurements in the laboratory are very controlled and do not necessarily correspond to real-world applications. Therefore, assessments in home-like environments are of crucial importance and a challenge when detecting gait impairments based on laboratory algorithms. Based on the lack of home-like validated algorithms, this paper presents a detection algorithm for HS and TO for home-like environments. The algorithm is first validated against an optoelectronic system during treadmill walking, in PD patients and older adults and second validated in an unconstrained environment using video footage. To the best of our knowledge, this is the first step detection algorithm using data obtained from an IMU at the lower back with very good accuracy, demonstrated across these divergent conditions in two different vulnerable populations and during different movement episodes.

## Methods

### Study Participants and Settings

#### Lab-Based Assessment

We performed the lab-based sub-study at the Robert Bosch Hospital, Stuttgart, Germany. It was approved by the ethics committee of the Medical Faculty at the University of Tübingen (protocol number 602/2012BO1). All participants provided written informed consent before they were included in the study. Patients were recruited from the outpatient clinic of the Neurology department at the University Hospital of Tübingen, Germany and were diagnosed by movement disorder specialists (Karin Srulijes and Walter Maetzler). Controls were recruited with the support of the office of Sport and Exercise and the Bosch BKK health insurance (Stuttgart, Germany). Exclusion criteria for both groups were inability to walk without walking aids for at least 20 m and the existence of additional neurological disorders. The analyses presented here are part of a larger study that focused on gait and eye movement interaction (28). The training group for the development of the algorithm consisted of three PD patients and two older adults who were randomly chosen from the dataset. The remaining participants (11 PD patients and 12 older adults) of the treadmill assessment were assigned to the test group. Table 1 provides demographic and clinical data of the two groups.

**Table 1:** Demographic and clinical data of the training and test groups.

	PD patients	Older adults
**Lab assessment**				
**Training cohort 1**		
*N* (females)	3 (2)	2 (1)
Age (years)	72.3 (4.7)	71.0 (2.8)
MDS-UPDRS III (0–132)	26 (15)	2 (2)
H&Y (0–5)	2 (1)	0 (0)
LED (mg)	640 (353)	0 (0)
**Test cohort 1**		
*N* (females)	11 (5)	12 (4)
Age (years)	74.7 (7.2)	70.8 (3.0)
MDS-UPDRS III (0–132)	39 (9)	1 (2)
H&Y (0–5)	3 (1)	0 (0)
LED (mg)	540 (298)	0 (0)
**Home-like assessment**		
**Training cohort 2**		
*N* (females)	4 (2)	2 (1)
Age (years)	69.3 (3.6)	63.0 (17.0)
MDS-UPDRS III (0–132)	20 (8)	1 (0)
H&Y (0–5)	2 (1)	0 (0)
LED (mg)	683 (735)	0 (0)
**Test cohort 2**		
*N* (females)	21 (11)	12 (6)
Age (years)	66.4 (9.0)	58.4 (8.9)
MDS-UPDRS III (0–132)	32 (12)	2 (4)
H&Y (0–5)	3 (1)	0 (0)
LED (mg)	841 (604)	0 (0)

*Data are shown as mean ± SD, except gender*.

*H&Y, Hoehn and Yahr; LED, Levodopa equivalent dose; PD, Parkinson’s disease; MDS-UPDRS III, motor part of the revised Unified PD Rating scale*.

All participants were equipped with 15 reflective markers [head (front, top, and back), trunk (jugulum, seventh cervical vertebra, and fifth lumbar vertebra), right/left arms, and right/left heel and toe; the “gold standard system”]. A six-camera Vicon T10 system (Vicon© Motion Systems Ltd., UK) (28) collected gait information from the reflective markers. The participants were also equipped with the Dynaport^®^ Hybrid, sample rate 100 samples/s, 3DOF accelerometer (range ±2 g), and 3DOF gyroscope (range ±100°/s) (McRoberts BV, Netherlands) at the lower back (the “experimental system”).

**Figure 1:**
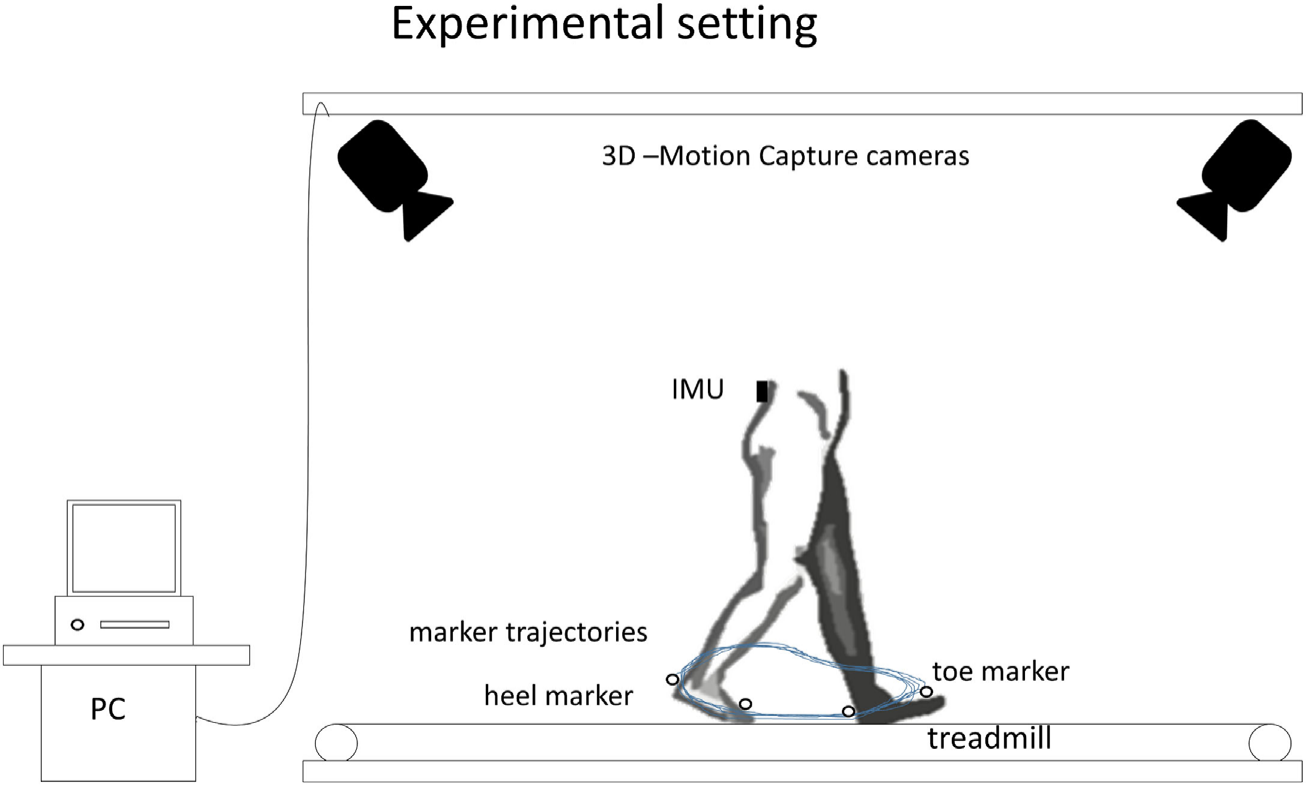
Experimental setting of the lab assessment showing the inertial measurement unit (IMU) position on the lower back, and the heel and toe markers to identify heel strike and toe off using the optoelectronic motion capture system.

During the 120s assessment, all participants walked on a treadmill (h/p/cosmos venus, h/p/cosmos sports medical GmbH, Germany) at their preferred speed. PD patients were tested ON medication. Manual markers set with the Dynaport^®^ Hybrid system at the beginning and the end of each 120s test period allowed *post hoc* synchronization of the gold standard and the experimental system. The comparison regarding the detection of step events from both systems was evaluated by one independent clinical observer (Morad Elshehabi). Figure 1 illustrates the experimental setting that was used in the lab assessment to provide a reliable HS and TO event detection. Treadmill walking guarantees regular straight walking, and the accuracy of the developed algorithm can be evaluated before being applied to more complex movements.

#### Home-Like Assessment

We performed the home-like sub-study at the University of Tübingen, Germany. This sub-study was approved by the ethics committee of the Medical Faculty at the University of Tübingen (protocol number 399/2012BO2). The study population consisted of a training group of 4 PD patients and 2 older adults, and a test group of 20 PD patients and 12 older adults. Four PD patients with challenging symptom constellations (e.g., tremor and dyskinesia) were chosen by Walter Maetzler, to increase specificity of the algorithm. Two older adults were randomly chosen from the database. The remaining participants of the home-like assessment were assigned to the test group. Patients were recruited from the inpatient and outpatient clinics of the Neurology department, University Hospital of Tübingen, and were diagnosed by a movement disorder specialist (Walter Maetzler). Exclusion criteria were deep brain stimulation, Hoehn and Yahr score >3 and Mini Mental State Examination score <24. Table 1 provides demographic and clinical parameters of the groups.

All participants were equipped with the OPAL system, sample rate 128 samples/s, 3DOF accelerometer (range ±16 g) and 3DOF gyroscope (range ±2,000°/s) (APDM, Inc., Portland, OR, USA). Data obtained from the IMU at the lower back were used for this analysis.

During the 180min (for PD patients) and 90min (for older adults) assessment, all participants were asked to perform daily-life activities such as moving around the labs and corridors, walking backwards, climbing stairs, performing transfers (sit-to-stand and stand-to-sit movements), making coffee, brushing teeth, and ironing clothes without any further restriction. During the whole process, one of the authors (Tanja Heger) followed the participant with a hand-held camera (Sony, resolution 1,920 × 1,080 pixels, frame rate 50 samples/s). Videos were evaluated by two clinical observers (Linda Haertner and Morad Elshehabi), to identify gait bouts, to label turning, and to count the number of steps per bout (29). We discarded those periods in which the feet of the participant were out of sight of the camera (7% of the total time of assessment).

### Algorithm Development and Structure (Training Groups)

**Figure 2:**
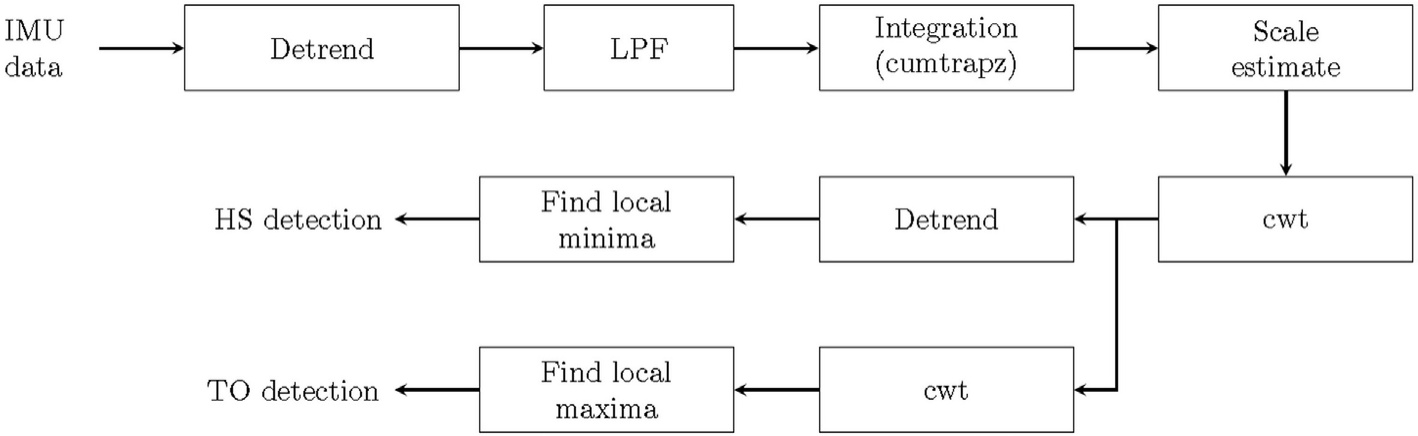
Heel strike (HS) and toe off (TO) detection using continuous wavelet transform (cwt) algorithm. IMU, inertial measurement unit; LPF, low-pass filter.

The algorithm was based on the continuous wavelet transform (cwt) approach. This was justified by previous studies that have shown very good results when extracting HS and TO events in healthy adults and PD patients under constrained condition (12, 30). From the lab-based assessment, HS and TO events were extracted from the anterior–posterior (AP) acceleration of the IMU and compared to the spatial signal of the heel and the toe markers of the Vicon system. From the unconstrained home-like assessment, we extracted only HS information, as the gold standard used in this study does not allow differentiating between HS and TO. Figure 2 provides the general structure of the algorithm.

#### Extraction of HS and TO from the Vicon System

**Figure 3:**
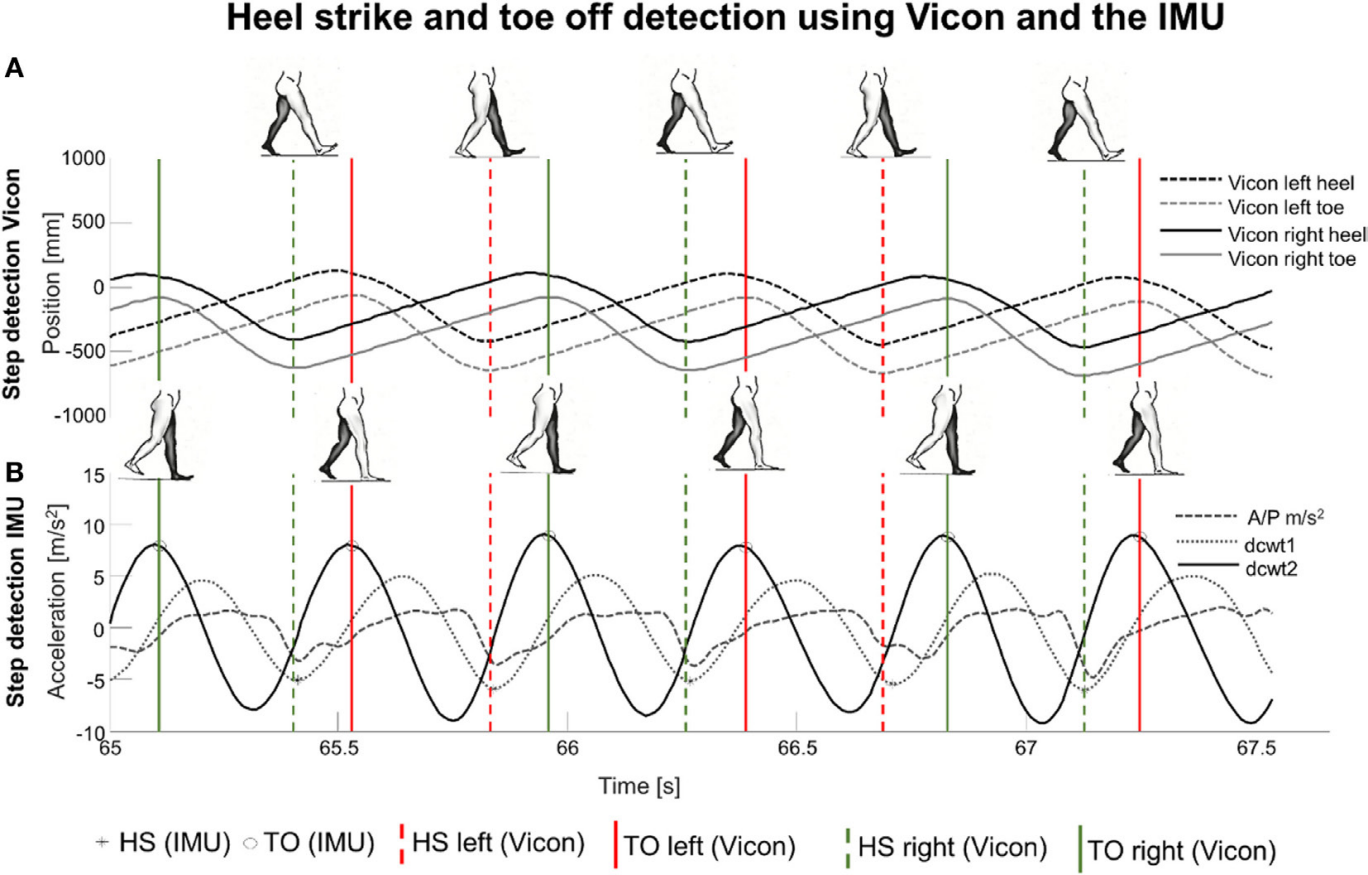
**(A)** Heel strike (HS) and toe off (TO) were detected from the anterior–posterior (AP) coordinates of the heel and toe markers (gold standard). **(B)** Event detection from both feet, from AP acceleration (dashed line), its first-order differential continuous wavelet transform (dcwt) (dotted line, dcwt1) and its second-order dcwt (solid line, dcwt2). Black stars indicate HS, and black circles indicate TO. Dashed and solid vertical lines enable the comparison of the two methods to detect HS and TO, showing an overall good correspondence between them.

HS and TO were extracted from the active Vicon markers from the left heel, right heel, left toe, and right toe. Details are provided in the legend of Figure 3. The bottom of the (left/right) heel marker curves, reflecting HS, and the top of the (left/right) toe marker curves, reflecting TO, were detected with the *findpeaks* Matlab function (Matlab R2015b).

#### Extraction of HS and TO from IMU

The algorithm for HS and TO detection from IMU data is illustrated in Figure 3 (12). The AP acceleration was preprocessed by linear de-trending and low-pass filtering at 10 Hz with a second-order Butterworth filter. The preprocessed signal was integrated (with *cumtrapz*) and differentiated by cwt (with the *cwt Matlab* function), using an estimated wavelet scale and Gaussian first-order (*gaus1*) wavelet.

The algorithm for wavelet scale estimation was based on the method by Abry (31). The most dominant frequency from the spectrum of the AP acceleration signal was selected and converted to the scale:

$$a=\frac{F_{c}}{F_{a}\cdot \Delta },$$

where *a* is the scale, *F_c_* is the center frequency (Hz) of the wavelet, *F_a_* is the most dominant frequency (Hz) (pseudo-frequency corresponding to the scale), and Δ is the sampling period (s).

Figure 3 illustrates results of HS and TO detection based on the Vicon and the IMU system. The local minima of the differentiated signal (first-order differentiated signal) were the detected HS points (with the *findpeaks* function). The first-order differentiated signal was differentiated again by similarly using cwt with estimated scale and Gaussian second-order (*gaus2*) wavelet, yielding a second-order differentiated signal. The local maxima of the latter signal were defined as TO (*findpeaks* function). The condition for the local extremes to be considered as HS/TO was as follows: magnitude >40% of the mean of all peaks detected by the *findpeaks* function.

#### Adaptation of the cwt to the Steps in the Home-Like Assessment

The IMU dataset for home-like assessment was split into two datasets, to evaluate step occurrence (1) during turning periods and (2) during non-turning periods. For the definition of turning periods, an algorithm recently published by our group was used (29). In brief, a change of the yaw angle (i.e., around the vertical axis) with a magnitude >90° and a duration between 0.5 and 10 s was defined as a turn.

As the shape of the integrated AP acceleration of usual steps differed slightly between the lab-based study and the home-like assessment (Figure 3), we adapted the wavelets in the home-like assessment as follows: We used a *gaus2* wavelet for step detection during turning periods and a Daubechies second-order (*db2*) wavelet for step detection during non-turning periods. Forty percent of the mean of all peaks detected by the *findpeaks* function served again as the threshold for step definition.

### Statistical Analysis

Analyses were performed with JMP 11.1.1 software. Mean and standard deviation (SD) were used to present demographic and clinical data of the groups.

For comparison of HS and TO detection, contingency tables were designed and χ^2^ tests conducted to test the relationship between methods and groups. Following the χ^2^ tests, the likelihood ratio (LR) was calculated to measure the association between the two methods.

As the dataset does not allow extracting “true negative steps,” we present total numbers of steps detected by the methods and accuracy values. True positive HS and TO from IMU were defined as <0.3 s difference relative to the respective Vicon event. Bland–Altman plots were created to evaluate the difference between the HS/TO events from the IMU and the HS/TO events from the gold standard.

For the analysis of the home-like assessment, intraclass correlation (ICC) was used to test the agreement of the step detection between the clinical observers. The ICC shows how likely a step was detected by the first clinical observer and also detected by the second clinical observer. We then calculated Cohen’s kappa, true positive, true negative, false positive, and false negative steps during turning and non-turning episodes (including straight walking, shuffling and walking backward episodes), respectively, from the IMU dataset based on the contingency tables. Cohen’s kappa yields the level of agreement between the steps detected by the algorithm and the steps detected by the clinical observers. In this dataset, we defined true negative steps as being below the step detection threshold (≤40%) introduced in the Section “Algorithm Development and Structure (Training Groups).”

## Results

### Validation

**Figure 4:**
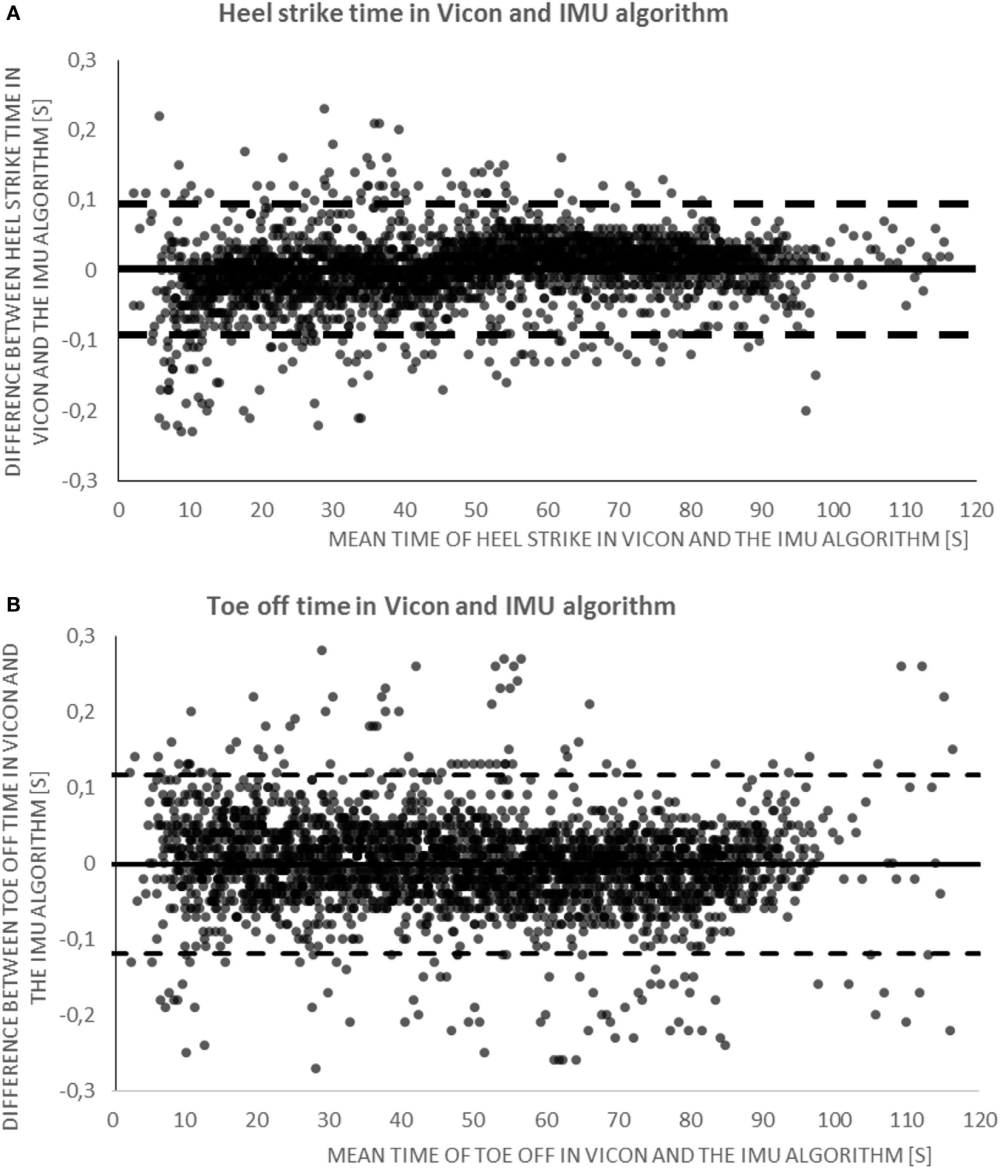
**(A)** Bland–Altman plot illustrating the agreement for time of heel strike detection between the algorithm and the gold standard. The continuous line is the mean, and dashed lines are the 95% confidence intervals (CIs) of step observation difference (in seconds). **(B)** Bland–Altman plot illustrating the agreement for time of toe off detection between the algorithm and the gold standard. The continuous line represents the mean, and dashed lines are the 95% CI of step observation difference (in seconds). IMU, inertial measurement unit.

In the lab assessment, for the whole group (which is presented here because no relevant differences occurred between the investigated groups; data available on request), the total number of HS/TO detected by Vicon was 2,730/2,739; by the IMU it was 2,729/2,732. Based on the 0.3s threshold, 9 HS/5 TO were considered false positives, and 10/12 false negatives. Accordingly, accuracies for HS/TO detection were 99/99% for all participants, 99/99% for PD patients, and 99/100% for older adults. LR calculated from χ^2^ was 0.8 and 0.83 for HS and TO, respectively. Bland–Altman plots showed a mean difference between IMU and Vicon of 0.00 s (95% CI, −0.09 to 0.10) for HS detection, and of 0.00 s (95% CI, −0.12 to 0.12 s) for TO detection (Figure 4).

In the home-like assessment, the ICC for step detection between clinical observers was 96%. During turning episodes, the total number of steps detected by clinical observers was 4,831; the IMU detected 5,020 steps. During non-turning episodes, the total number of steps detected by clinical observers was 19,885, and from IMU data it was 21,313. During turning episodes, we obtained accuracies for step detection by IMU of 88% for PD patients and 89% for older adults. The corresponding accuracy values for non-turning episodes were 91 and 91%, respectively. Table 2 provides detailed information about the validation values for these analyses.

**Table 2:** Validation values for steps detection in the home-like assessment.

Cohorts	Cohen’s kappa (%)	Acc (%)	Sens (%)	Spec (%)	NPV (%)	PPV (%)	Steps detected by algorithm	Steps detected by clinical observers	True positive steps	False positive steps	False negative steps	True negative steps
**Turning episodes**												
Overall cohort	73	88	90	85	78	94	5,020	4,831	4,517	314	503	1,730
PD patients	70	88	90	83	75	94	3,618	3,484	3,257	227	361	1,083
Controls	77	89	90	88	82	94	1,402	1,347	1,260	87	142	647
**Non-turning episodes**												
Overall cohort	72	91	91	90	69	98	21,313	19,885	19,429	456	1,884	4,096
PD patients	71	91	91	90	67	98	15,522	14,493	14,181	312	1,341	2,738
Controls	74	91	91	90	71	97	5,791	5,392	5,248	144	543	1,358

*Acc, accuracy; NPV, negative predictive value; PD, Parkinson’s disease; PPV, positive predictive value; Sens, sensitivity; Spec, specificity*.

## Discussion

We present in this paper an algorithm for step detection during non-turning walking episodes on a treadmill and during turning and non-turning walking episodes in a home-like assessment, using a single IMU worn at the lower back. The algorithm was tested in PD patients and older adults and yielded very good accuracy. It might thus provide a valuable tool for future daily-life-like assessments of gait in these cohorts.

One of the major advantages of the algorithm is the consideration of steps during turning. Assessment of steps during turning episodes is frequently omitted in current research (12, 23). Although the accuracy values for step detection during turning were slightly lower than during straight walking episodes (88% compared to 91%), results are still promising and indicate the suitability of the algorithm for gait assessment in daily-life conditions. Moreover, the algorithm showed high accuracy during non-turning episodes, which included stepping on the spot and walking backwards.

This algorithm is reliable in both, lab assessments and home environment, with the adaptation of the used wavelet types. We think that such adaptation of algorithms can substantially increase their validity, and even might be promising when used at an individual level. Such approaches have already been introduced in commercially available step detection systems used in the health and fitness sector, and validated for questionnaire-based assessment in diseases such as cerebral palsy (32, 33). Previous studies demonstrated the frequent challenge to compare results from the lab versus the home environments, as the patients behave differently and show more complex movements in the home environment (18, 34–36). The reliability of the current algorithm in home-like environment indicates its applicability in future assessments of PD patients and older adults in more natural unobtrusive surroundings.

The higher accuracy of the algorithm in the lab assessment versus the home-like environment might not only be due to more regular and “simpler” stepping patterns on the treadmill compared to free living-like movements, but also to the higher accuracy of the gold standard in the lab assessment [Vicon (37, 38) versus video observation, visual classification of a step belonging to a turn or non-turn episode]. Assessment based on clinical observers bears several limitations, e.g., that clinical observers can inaccurately define a step as part of a turning episode (39).

Our algorithm showed comparable validity in our average moderately affected PD patients and in older adults. This finding implies that motor impairments, in particular gait deficits, do not limit the reliability of our algorithm given that individuals can walk without aid. This aspect may be essential to the applicability of the algorithms in different healthy and pathological conditions. Previous algorithms used the vertical acceleration for step detection (12, 30). However, we opted to applying the AP acceleration, which is also an established method, already used in previous studies (23, 40, 41). The vertical acceleration may have better accuracy, but it yielded a more complex pattern in our training dataset in particular in the home-like environment data, which reduced the signal’s regular consistency (40).

The following limitations should be considered in future studies and algorithm development. In the home-like assessment, the gold standard (videotaping) allowed only the validation of step occurrence, but not of HS and TO. Therefore, it was not possible to validate qualitative step parameters (for example, step time, stance time, and swing time), although the algorithm provides these data. Based on the very good results from the treadmill evaluation, we are optimistic that HS and TO parameters extracted from the algorithm in the home-like assessment would also be valid. This hypothesis has to be evaluated in future studies using, e.g., instrumented shoes and insoles. Instrumented shoes with feet IMUs (23) and instrumented insoles (42) provide HS and TO event detection and can be used in home-like conditions (23, 42). Another limitation is the choice of wavelet types and thresholds for step detection. We made these choices of the wavelet types and threshold based on visual inspection of the AP acceleration in the training groups. It is still possible that other wavelet shapes and threshold values yield even better accuracy in these populations and activities. Furthermore, it is worth noting that the error of 0.1 s in HS and TO detection was not negligible if we want to estimate stride time variability. Although some variation may be explained by physiological differences (e.g., feet and lower back may not always be synchronized), further efforts should be undertaken to reduce this variation and increase the sample size to improve the statistical power and reduce the error of such outliers. Moreover, the sample size could be expanded in future studies to test the validity of the algorithm in a bigger cohort.

This study presents and validates an algorithm for step detection during treadmill walking, during turning and non-turning walking episodes based on data extracted from an IMU at the lower back, for PD patients and older adults. The algorithm was tested with different validation methods: optical markers and videotaping. While the results are promising, future work has to investigate the validity of the algorithm in different disease phases of PD including the prodromal phase and potentially also phases in which patients use walking aids.

## Ethics Statement

This study was carried out in accordance with the recommendations of Medical Faculty at the University of Tübingen, protocol number 602/2012BO1, with written informed consent from all subjects. All subjects gave written informed consent in accordance with the Declaration of Helsinki. The protocol was approved by the Medical Faculty at the University of Tübingen.

## Author Contributions

MP and WM were responsible for the conception and design of the study. KS, TH, MS, CB, and WM contributed to acquisition of data. MP, ME, LH, SD, LR, GS, and WM were involved in analysis and interpretation of data. MP and WM drafted the first version of the article. MP, ME, LH, SD, KS, MS, MH, GF, CH, DS, JF, DB, AS-F, JD, CB, LR, GS, and WM revised it critically for important intellectual content. All the authors gave final approval of the version to be submitted.

## Conflict of Interest Statement

The authors declare that the research was conducted in the absence of any commercial or financial relationships that could be construed as a potential conflict of interest. The reviewer, FC, and handling editor declared their shared affiliation.
